# Determine the prevalence of gestational diabetes in Ardakan and its related factors

**DOI:** 10.1016/j.mex.2019.02.016

**Published:** 2019-02-27

**Authors:** Ahmad Moradi, Hamid Reza Morovati, Amir Teimourpour, Shahrzad Nematollahi, Monireh Faghir Ganji

**Affiliations:** a Department of Public Health, Shoushtar Faculty of Medical Sciences, Shoushtar, Iran; b Department of Epidemiology and Biostatistics, School of Public Health, Shahid Sadoughi University of Medical Sciences, Yazd, Iran; c Department of Epidemiology and Biostatistics, School of Public Health, Tehran University of Medical Sciences, Tehran, Iran; d Students’ Scientific Research Center, Tehran University of Medical Sciences, Tehran, Iran

**Keywords:** Gestational diabetes, Glucose tolerance test, Yazd Province

## Abstract

Gestational Diabetes (GD) is amongst the most common metabolic disorders. Due to the important complications of GD on maternal and fetal health and in order to identify the prevalence of GD in various climate and cultures, the present studies aimed to determine the prevalence of GD in Ardakan and its related factors in 2014–2015. This cross-sectional study was conducted in 3808 pregnant women referring to rural and urban health centers in Ardakan city in 2013–14. Demographic, clinical, and obstetrics history of the subjects was gathered. GD was defined based on Glucose Tolerance Test (GTT). Descriptive and Logistic regression models were applied. The prevalence of GD was estimated to be 7.5% (286) which was higher in 35–39 age group, urban residents, obese mothers, and pregnancies ended with macrosome babies. The odds of GD was higher in obese mothers by 1.62 times (95%CI: 1.18–2.24), in mothers above ages of 40 by 10.53 (95%CI: 3.8–29.3), in mothers with a history of GD by 3.86 (95%CI:1.65–8.93), and in pregnancies ended with a macro some baby by 2.2 (95%CI: 0.97–5.1). The prevalence of GD in Ardakan was similar to other studies in the area. It seems that improvement of GD screening in older mothers and those with a history of GD could be a priority of surveillance system in Yazd Province.

## Graphical abstract

**Figure:**
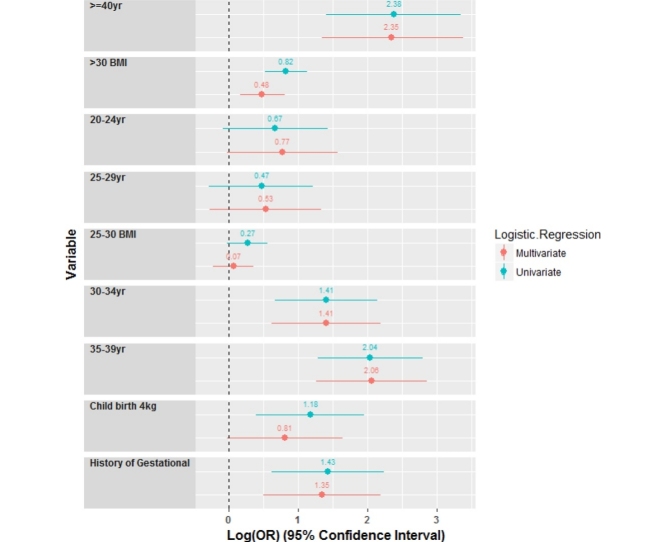

**Specifications Table**

**Table:** 

**Subject area:**	Gestational Diabetes (GD)
**More specific subject area:**	Common Metabolic Disorders
**Protocol name:**	Determine the prevalence of Gestational Diabetes
**Reagents/tools:**	Demographic, clinical, and obstetrics history of the subjects was gathered. GD was defined based on Glucose Tolerance Test (GTT). Descriptive and Logistic regression models were applied
**Experimental design:**	This descriptive-analytic cross-sectional study has been conducted on the information of all pregnant women including 4083 women who were referred to Comprehensive Health Services Centers and Healthcare Centers in Ardakan in 2014 for receiving care of pregnancy
**Trial registration:**	No applicable
**Ethics:**	No applicable

## Protocol data

• Our study showed that the highest prevalence of gestational diabetes in women aged 35–39 years.
• The odds of gestational diabetes increase significantly with increasing maternal age, body mass index (BMI), gestational diabetes in previous pregnancies and macrosomal child.
• Planning for effective prevention and intervention at local and provincial levels, with an emphasis on the food culture and customs of each region, can increase the health of mothers and infants.

## Description of protocol

**Fig. 1:**
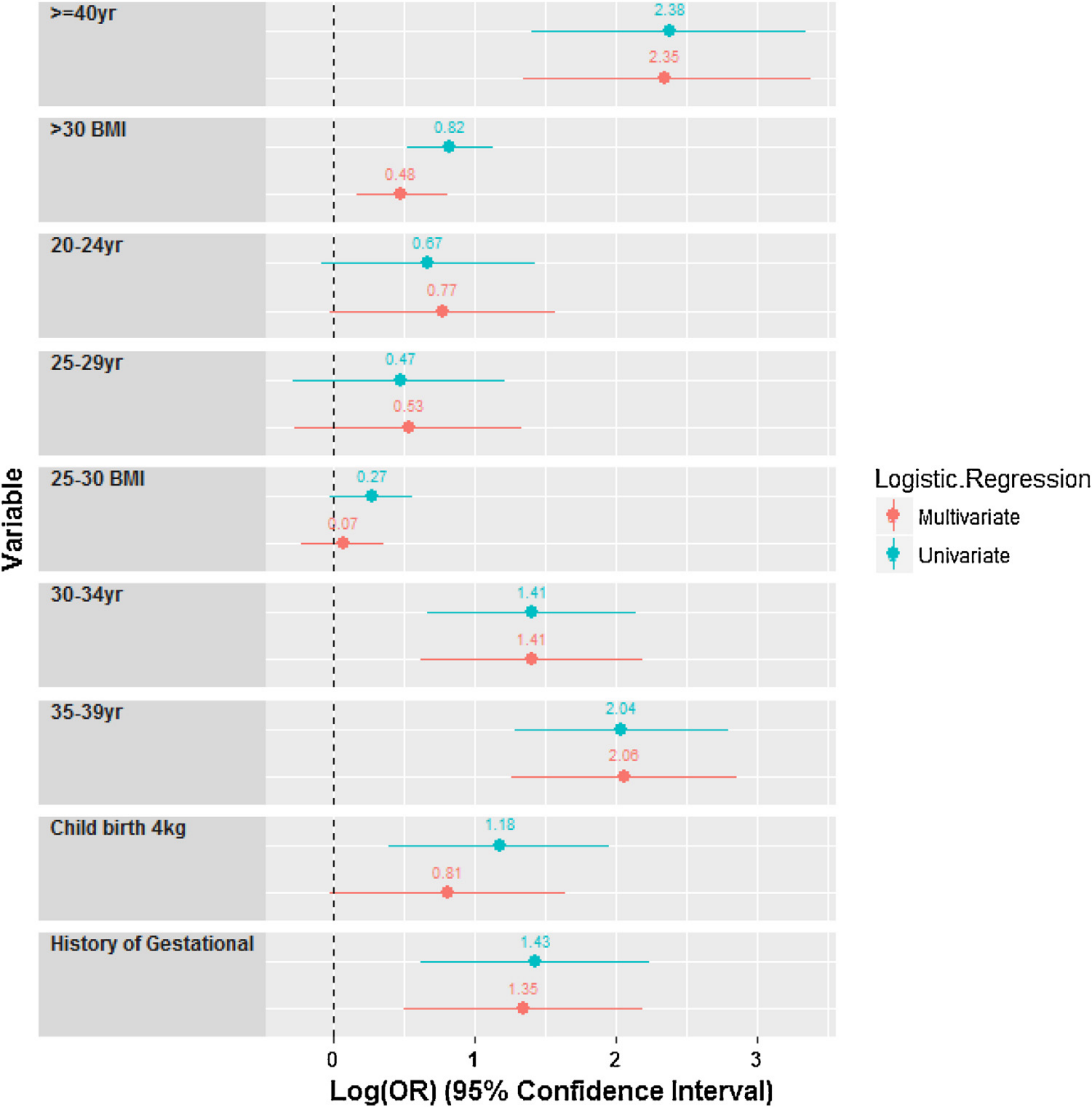
The forest plot of adjusted and unadjusted regression mod.

**Fig. 2:**
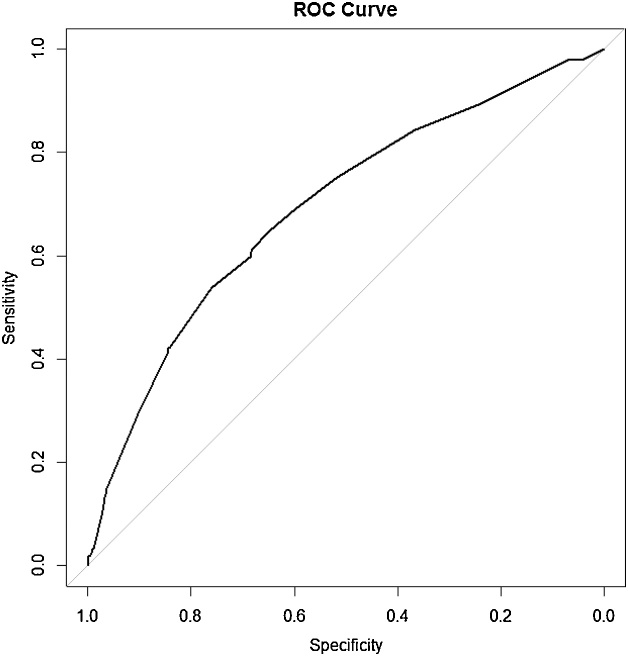
ROC curve of final model with 4 independent variables.

**Fig. 3:**
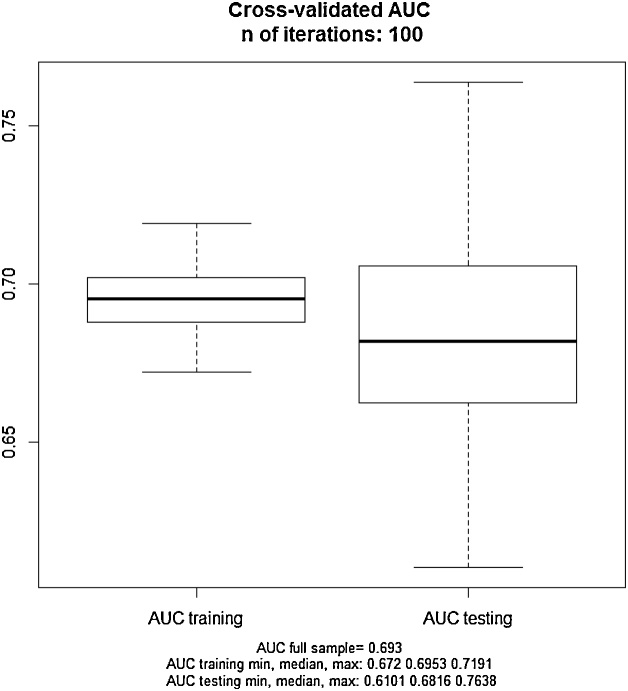
Boxplot of validation procedure.

Among 3808 pregnant women studied, 286 (7.5%) women were diagnosed with gestational diabetes, the highest prevalence of gestational diabetes in women aged 35–39 years (64, 18.4%). The mean age was 27.1 (5.4) with a range of 14–46 years, and the mean body mass index (BMI) was 25.7 (4.9). The mean gestational age was 10.7 (6.6) weeks with a range of 3–34, 93 women (2.4%) were rural residents and 3715 (97.6%) women were urban residents. The mean number of pregnancies was 2.14 with a range of 1–8. Body mass index was divided into three groups: normal (less than or equal to 24.9) and overweight (25–29.9) and obese (more than or equal to 30). Accordingly, 1716 women were in the normal weight group (45.6%), 1348 women were in the overweight group (35.8%) and 702 women were in the obese group (18.6%). In obese women, GDM (gestational diabetes mellitus) of 85 women (12.1%) was more, and in pregnancy that resulted in abortion, 18 women (9%) had a slightly higher prevalence of gestational diabetes Approximately 1.4% of mothers with a birth weight of over 4 kg had GDM, as well as the average pregnancy rank (2.48%) was higher in the mothers with GDM than other mothers (2.12%). The distribution of GDM incidence based on age groups is presented in Table 1. The results of univariate logistic regression analysis are given in Table 2. In the following, regarding the method of Hosmer–Lemeshow to develop the final logistic regression model, the results are reported in Table 3. Also, Fig. 1 shows Forest plot of adjusted and unadjusted logistic regression models in the form of an algorithm. Considering insignificance of goodness test for the fitting of Hosmer–Lemeshow (chi square = 5.406, df = 8, P-value = 0.713) yields the good fit of the multiple regression logistic model. The rock curve derived from the multiple logistic regression with the area under the curve of 0.693 (95% CI 0.66–0.726) is shown in Fig. 2. Using Youden index, the best cut off for multivariate logistic regression models was 0.297 with a sensitivity of 0.649 and feature of 0.648. Finally, Fig. 3 shows the boxplot of the results of the evaluation of multivariate regression model using Giancristofaro method. We can conclude, based on the two boxplots, the validity of multivariate regression model. According to the analysis of Multivariate Logistic Regression Model, the mothers with a BMI greater than 30 had 62% (significantly) higher odds of GDM. The odds of GDM in pregnancy that leads to a baby above 4 kg is 2.2 times more than other pregnancies (P = 0.05) (95% CI: 0.979–5.137).

**Table 1:** The distribution of GDM incidence based on age groups.

			age 1						Total
			>19	20–24	25–29	30–34	35–39	<40	
event	GDM	Count	8	54	56	93	64	11	286
		% within event	2.8%	18.9%	19.6%	32.5%	22.4%	3.8%	100.0%
	Healthy	Count	274	945	1204	780	284	35	3522
		% within event	7.8%	26.8%	34.2%	22.1%	8.1%	1.0%	100.0%
Total		Count	282	999	1260	873	348	46	3808
		% within event	7.4%	26.2%	33.1%	22.9%	9.1%	1.2%	100.0%

**Table 2:** The results of univariate analysis for gestational diabetes mellitus according to the characteristics of pregnant mothers referring to health centers in Ardakan during 2014–2015.

Variable		GDM	Number	Percentage	P-value*
BMI	<=25	Total	1716	45.6	<0.001
		GDM	98	34.8	
		Not GDM	1618	46.4	
	25–30	Total	1348	35.8	
		GDM	99	35.1	
		Not GDM	1249	35.8	
	>=30	Total	702	18.6	
		GDM	85	30.1	
		Not GDM	617	17.7	
Age	=<19	Total	282	7.4	<0.001
		GDM	8	2.8	
		Not GDM	274	7.8	
	20–24	Total	999	26.2	
		GDM	54	18.9	
		Not GDM	945	26.8	
	25–29	Total	1260	33.1	
		GDM	56	19.6	
		Not GDM	1204	34.2	
	30–34	Total	873	22.9	
		GDM	93	32.5	
		Not GDM	780	22.1	
	35–39	Total	348	9.1	
		GDM	64	22.4	
		Not GDM	284	8.1	
	>=40	Total	46	1.2	
		GDM	11	3.8	
		Not GDM	35	1	
Family history of diabetes	No	Total	2825	74.2	<0.001
		GDM	182	63.6	
		Not GDM	2643	75	
	Yes	Total	983	25.8	
		GDM	104	36.4	
		Not GDM	879	25	
History of Gestational diabetes	No	Total	3776	99.2	0.002
		GDM	278	97.2	
		Not GDM	3498	99.3	
	Yes	Total	32	0.7	
		GDM	8	2.8	
		Not GDM	24	0.8	
History of abortion	No	Total	3707	97.3	0.116
		GDM	274	95.8	
		Not GDM	3433	97.5	
	Yes	Total	101	2.7	
		GDM	12	4.2	
		Not GDM	89	2.5	
The birth of a baby above 4 kg	No	Total	3769	99	0.009
		GDM	278	97.2	
		Not GDM	3491	99.1	
	Yes	Total	39	1	
		GDM	8	2.8	
		Not GDM	31	0.9	
Residential	Urban	Total	93	2.4	0.199
		GDM	4	1.4	
		Not GDM	89	2.5	
	Rural	Total	3715	97.6	
		GDM	282	98.6	
		Not GDM	3433	97.5	
History of Pre-diabetic	No	Total	3796	99.7	0.292
		GDM	284	99.3	
		Not GDM	3512	99.7	
	Yes	Total	12	0.3	
		GDM	2	0.7	
		Not GDM	10	0.3	
History of hypertension	No	Total	3749	98.5	0.781
		GDM	281	98.3	
		Not GDM	3468	98.5	
	Yes	Total	59	1.5	
		GDM	5	1.7	
		Not GDM	54	1.5	
The pregnancy rank	First	Total	1206	31.7	<0.001
		GDM	65	22.7	
		Not GDM	1141	32.4	
	Second	Total	1395	36.7	
		GDM	82	28.7	
		Not GDM	1313	37.3	
	Third	Total	812	21.3	
		GDM	91	31.8	
		Not GDM	721	20.5	
	More than third	Total	393	10.3	
		GDM	48	16.8	
		Not GDM	345	9.8	

* Omnibus Test of Univariate logistic Regression Coefficients (likelihood ratio test).

**Table 3:** The results of multivariate analysis for gestational diabetes according to the characteristics of pregnant mothers referring to health centers in Ardakan during 2014–2015.

	Univariate			Multivariate		
Variable	OR	95%CI	P-Value*	OR	95%CI	P-Value*
BMI			<0.001			0.006
<25	Reference			Reference		
25–30	1.309	0.98–1.747	0.068	1.07	0.795–1.44	0.655
>30	2.275	1.677–3.085	<0.001	1.624	1.18–2.241	0.003
Age			<0.001			<0.001
=<19	Reference			Reference		
20–24	1.957	0.92–4.162	0.081	2.154	0.967–4.795	0.06
25–29	1.593	0.751–3.380	0.225	1.704	0.767–3.786	0.191
30–34	4.084	1.958–8.518	<0.001	4.09	1.868–8.97	<0.001
35–39	7.718	3.633–16.4	<0.001	7.85	3.515–17.52	<0.001
>=40	10.764	4.055–28.57	<0.001	10.53	3.87–29.33	<0.001
History of Gestational						
No	Reference			Reference		
Yes	4.194	1.867–9.423	0.001	3.843	1.654–8.93	0.002
The birth of a baby above 4 kg						
No	Reference			Reference		
Yes	3.241	1.476–7.117	0.003	2.243	0.979–5.137	0.056

* Wald test.

## Materials and methods

This descriptive-analytic cross-sectional study has been conducted on the information of all pregnant women including 4083 women who were referred to Comprehensive Health Services Centers and Healthcare Centers in Ardakan in 2014 for receiving care of pregnancy. The information used in the present study included demographic and clinical information of the mother (such as BMI in the three groups of normal (less than or equal to 24.9), overweight (25–29.9) and obese (more than or equal to 30), maternal fertility history (successful and unsuccessful pregnancy, abnormal birth weight, pregnancy and delivery rank, history of abortion and stillbirth, and history of complications of childbirth), and the condition of the mother with all types of diabetes [[1], [2], [3], [4], [5]]. The risk factors for gestational diabetes such as over age 30, gestational diabetes history in previous pregnancies, history of pre-diabetes (IFG (impaired fasting glucose) or IGT (impaired glucose tolerance)), history of diabetes in the first-degree family, high blood pressure history, birth history of 4 kg or more, BMI equal to or greater than 30 kg/m² before pregnancy, history of spontaneous abortion with no cause twice or more times, history of stillbirth, history of fetal abnormalities in previous pregnancies were cases used in the present study.

Also, gestational diabetes is defined as high blood sugar levels three hours after consuming 75 g of glucose for women with a fasting blood sugar level greater than 92–126 mg/dl and at the 24th to 28th gestational weeks [6]. According to this definition, the mothers with type 2 diabetes were excluded from the study. Also, some of the information of the pregnant mother, such as the end of pregnancy (newborn, abortion, and stillbirth), delivery type (natural or cesarean), gestational age, birth weight, baby abnormalities, maternal complications (prolonged delivery and delivery bleeding) were also used for the present study. The data were analyzed using software SPSS (version 24). The main consequence of this study was the classification of gestational diabetes, which is as a two-state variable (yes/no), and in addition to a descriptive analysis, univariate and multivariate logistic regression model was used for obtaining modified effects of risk factors as the odds ratio (OR). The criterion used for independent variables in the multiple logistic regression model was also considered to be less than p value of 0.2 in all analyzes, the level of significance was less than 0.05 Software SPSS version 21 was used to analyze the data.

### Statistical methods

Descriptive statistics have been reported for quantitative variables including mean and standard deviation, and for qualitative variables including frequency (%). In order to study the unadjusted effect of each of the independent variables (risk factors) on the incidence of disease, one-variable logistic regression model analysis was used. In order to obtain adjusted data for each independent variable according to Hosmer and Lemeshow approach to develop multivariate logistic regression model [7], those variables with a likelihood ratio test of less than 0.2 were introduced into multivariate logistic regression model and adjusted effects’ estimation was reported according to the method of Hosmer and Lemeshow. Also, in order to study the goodness of fitting of the regression model, the goodness test of the fit of Hosmer and Lemeshow was used. In order to study the differential power of the logistic regression model, Receiver Operating Characteristic (ROC) curves and the area under the curve (AUC) were used [8]. Meanwhile, in order to obtain the best cut-off point for the estimated possibility values by the final multivariate logistic regression model Youden index was used. Finally, in order to evaluate the validity of the final logistic regression model, the method described in [9] has been used, based on this method, first the data were randomly assigned 100 times to two parts with ratios of 75 and 25 percent, then fitted using the larger part (75%) of the model and the model parameters are estimated. Then, using the smaller part data (25%) and estimated parameters at the previous stage, response variable values were predicted, ROC curves are plotted and AUC was provided for both parts of the data. Also, forest plot was used to show adjusted and unadjusted effects of each of the independent variables (risk factors) on the logarithmic scale [10]. All statistical and analytical calculations were performed by software R version 3.5.0 (32bit) and using packages of ggplot2, CA Tools and pROC.

## Conflict of interest

All authors declare that they have no conflict of interest.
